# Tunable Electronic Bandgaps and Optical and Magnetic Properties in Antiferromagnetic MPS_3_/GaN (M = Mn, Fe, and Ni) Heterobilayers

**DOI:** 10.3390/nano15110832

**Published:** 2025-05-30

**Authors:** Shijian Tian, Li Han, Libo Zhang, Kaixuan Zhang, Mengjie Jiang, Jie Wang, Shiqi Lan, Xuyang Lv, Yichong Zhang, Aijiang Lu, Yan Huang, Huaizhong Xing, Xiaoshuang Chen

**Affiliations:** 1 The College of Physics, Donghua University, Shanghai 201620, China; 2181615@mial.dhu.edu.cn (S.T.); 2191781@mail.dhu.edu.cn (M.J.); wangjie@mail.dhu.edu.cn (J.W.); lsh18805887497@163.com (S.L.); 2212318@mail.dhu.edu.cn (X.L.); yichongzhang0000@163.com (Y.Z.); ajlu@dhu.edu.cn (A.L.); 2 College of Physics and Optoelectronic Engineering, Hangzhou Institute for Advanced Study, University of Chinese Academy of Sciences, No. 1, Sub-Lane Xiangshan, Xihu District, Hangzhou 310024, China; zhanglibo@ucas.ac.cn (L.Z.); zhangkaixuan@ucas.ac.cn (K.Z.); 3 College of Optical and Electronic Technology, China Jiliang University, Hangzhou 310018, China; 4 State Key Laboratory of Infrared Physics, Shanghai Institute of Technical Physics, Chinese Academy of Sciences, Shanghai 200083, China; yhuang@mail.sitp.ac.cn (Y.H.); xschen@mail.sitp.ac.cn (X.C.)

**Keywords:** two-dimensional materials, MPS_3_, antiferromagnetism, heterobilayer, first-principles calculations, optoelectronics, spintronics

## Abstract

Research on two dimensional (2D) antiferromagnetic materials and heterobilayers is gaining prominence in spintronics. This study focuses on MPS_3_ monolayers and their van der Waals heterobilayers with GaN monolayers. We systematically investigated the structural stability, electronic properties, and magnetic characteristics of MPS_3_ (M = Mn, Fe, and Ni) monolayers via first-principles calculations, and explored their potential applications in optoelectronics and spintronics. Through phonon spectrum analysis, the dynamic stability of MPS_3_ monolayers was confirmed, and their bond lengths, charge distributions, and wide-bandgap semiconductor properties were analyzed in detail. In addition, the potential applications of MPS_3_ monolayers in UV detection were explored. Upon constructing the MPS_3_/GaN heterobilayer structure, a significant reduction in the bandgap was observed, thereby expanding its potential applications in the visible light spectrum. The intrinsic antiferromagnetic nature of MPS_3_ monolayers was confirmed through calculations, with the magnetic moments of the magnetic atoms M being 4.560, 3.672, and 1.517, respectively. Moreover, the heterobilayer structures further enhanced the magnetic moments of these elements. The magnetic properties of MPS_3_ monolayers were further analyzed using spin-orbit coupling (SOC), confirming their magnetic anisotropy. These results provide a theoretical basis for the design of novel two-dimensional spintronic and optoelectronic devices based on MPS_3_.

## 1. Introduction

In 2004, Novoselov and Geim investigated graphene’s monolayer structure, confirming the possibility of exfoliating stable single-atom or single-polyhedron thickness two dimensional (2D) materials from van der Waals solids [1,2,3]. Since then, there has been sustained enthusiasm for exploring layered van der Waals materials, with their unique and fascinating physical properties gaining attention. Many materials that can be exfoliated and studied in the 2D limit have been discovered or rediscovered [4,5]. Building on this, the discovery of magnetic ordering phenomena in 2D van der Waals materials at the 2D limit has made it possible to systematically study the effects of dimensionality on magnetic ordering and to integrate magnetic materials into van der Waals heterostructures [6,7,8,9]. Long-range magnetic order has been observed in materials such as Cr_2_Ge_2_Te_6_, Fe_3_GeTe_2_, and CrI_3_. Moreover, many inherently non-magnetic two-dimensional materials, such as graphene, hexagonal BN, and transition metal dichalcogenides, can exhibit magnetic properties through defect engineering, strain manipulation, and chemical modifications. This avenue of research holds promise for expanding the scope of magnetic functionalities in 2DM and advancing spintronic applications. Recently, scientists have focused their attention on exploring and utilizing many different magnetic states, including ferromagnetic (FM) and antiferromagnetic (AFM) ground states, while controlling magnetism through external stimuli such as strain engineering, defects, and electric fields, and modifying the arrangement of the magnetocrystalline arrays, leading to the discovery of compelling physical phenomena. Interestingly, although AFM states are internally magnetic, their net magnetization is zero, which may lead to a variety of remarkable phenomena in 2D confinement [10]. Furthermore, combining electrical, optical, and magnetic properties can lead to mesmerizing magnetoelectric or magneto-optical coupling effects [11,12].

Intralayer AFM materials encompass a diverse array of compounds that have garnered increasing interest. Leading the pack in this category are transition metal thiophosphates MPS_3_ (M = Mn, Fe, Co, or Ni) and selenophosphates MPSe_3_ (M = Mn, Fe, or Ni), where layered AFM has been meticulously examined and confirmed through various experimental methodologies. FePS_3_, CoPS_3_, NiPS_3_, and FePSe_3_ exhibit a zigzag-type AFM arrangement, while MnPS_3_ and MnPSe_3_ demonstrate Néel-type ordering [13,14,15,16]. Further intricacies emerge in the cases of FePS_3_, FePSe_3_, and MnPS_3_, which feature out-of-plane anisotropy, contrasting with the dominant in-plane anisotropy observed in NiPS_3_ and MnPSe_3_. Previous investigations have revealed that the bulk form of these materials with M = Mn, Fe, and Ni exhibit AFM behavior, with Neel temperatures measured at 78 K, 123 K, and 155 K, respectively [17]. Additionally, studies have delved into the metal-to-insulator transition and the emergence of superconducting phases within this family of compound [18,19,20]. These unique properties make MPS_3_ family materials promising candidates for a wide range of applications in the fields of electronics, spintronics, and quantum technologies. Further research into their fundamental properties and potential technological applications is warranted to fully harness their capabilities. Recent reports have demonstrated an all-optical control of the magnetic anisotropy in NiPS_3_ by tuning the photon energy in resonance with an orbital transition between crystal field-split levels. This finding opens up a new application direction for utilizing the potential of transition metal phosphorus trichalcogenides in the field of optical control, paving the way for new possibilities in areas such as optoelectronics and information storage technologies.

Understanding and controlling surfaces is crucial for designing and developing new materials with specific properties and functionalities. The integration of two or more types of 2D van der Waals heterobilayers (vdWHs) along the vertical direction introduces novel properties [21,22,23,24,25,26,27,28,29,30,31]. In this work, considering all the superior properties of both MPS_3_ (M = Mn, Fe, and Ni) and GaN nanosheets, we constructed the MPS_3_/GaN vdWHs (in the following text, the MPS_3_/GaN vdWHs will be uniformly abbreviated as vdWHs) and investigate their electronic, magnetic, and optical properties using DFT. The work is organized as follows: a brief overview of computational information is provided in Section 2; detailed electronic, magnetic, and optical properties for MPS_3_ MLs and vdWHs are discussed in Section 3. Finally, a summary is provided in Section 4.

## 2. Computational Information

All DFT calculations were performed by using the projector-augmented wave (PAW) [32] pseudopotential, as implemented in the Vienna ab initio Simulation Package (VASP) [33,34]. For structural optimizations and energy calculations, the exchange-correlation potential based on the Perdew–Burke–Ernzerhof function with a generalized gradient approximation (PBE-GGA) was used [35,36]. To make sure all geometric structures were fully relaxed, the energy and force convergence on each atom were set to 10^−5^ eV/atom and 0.001 eV/Å, respectively. The Monkhorst–Pack method with 5 × 5 × 1 K-points meshes of Brillouin zones was selected for geometric relaxation and 15 × 15 × 1 k-mesh was used for calculating the density of states (DOS). Moreover, the Hubbard-like Coulomb interaction U and the Screened Stoner exchange parameter J are considered. The effective parameter is defined as U_eff_ = U − J and applied on the M (Mn, Fe, and Ni) 3d orbitals, and has been set to 4.0, 4.6, and 5.1 eV, respectively. These values have been studied before [37]. The DFT-D3 method was adopted to address the issue of inaccurate dispersion description by PBE [38]

## 3. Results and Discussion

### 3.1. Structural Properties and Stability

**Figure 1:**
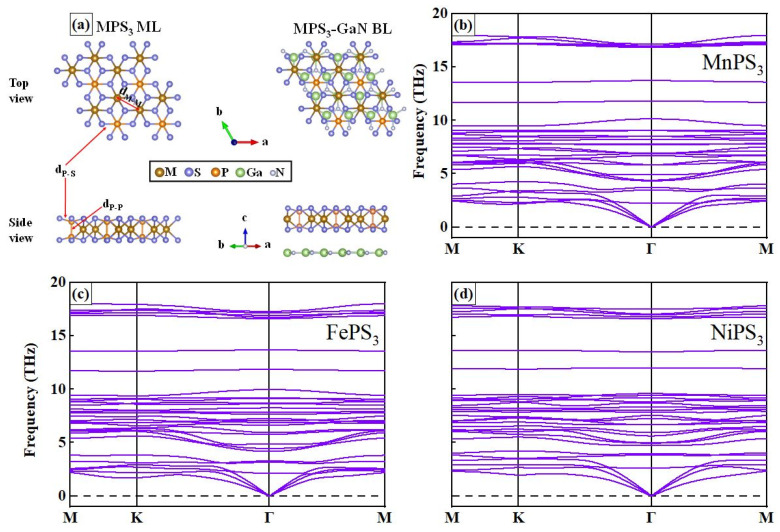
(**a**) The top and side view in atomic level for MPS_3_ MLs and vdWHs, the phonon dispersion for (**b**) MnPS_3_, (**c**) FePS_3_, and (**d**) NiPS_3_ MLs.

The top and side views of the atomic structures of free-standing GaN MLs was relaxed to a flat honeycomb structure which was stripped from the (0001) plane of a wurtzite GaN structure (which has been analyzed before) [39]. MPS_3_ belongs to layered vdWs materials, as shown in Figure 1a, and M atoms are sandwiched by P- and S-constituted planes. We first calculated the energy difference of the system (△E = E_AFM_ − E_FM_) and presented the results in Table 1 and Table S2. The negative values for both MPS_3_ MLs and vdWHs indicate that the ground state of the three MPS_3_ MLs is AFM. The persistence of the AFM state in the vdWHs also indicates that the interfacial interactions do not significantly disrupt the magnetic ordering, which is an important consideration for designing layered materials with tailored properties.

As shown in Table 1, the calculated lattice parameters of GaN, MnPS_3_, FePS_3_, NiPS_3_ MLs are a = b = 6.38 Å, 6.13 Å, 6.00 (a ≈ b) Å, and 5.89 Å, respectively (as shown in Table 1). The lattice mismatch between MPS_3_ and GaN MLs were calculated to 4.07%, 6.33%, and 8.32%, respectively. Based on these data, it can be observed that the lattice mismatches of MnPS_3_, FePS_3_, and NiPS_3_ MLs are all below 10%. This indicates that the lattice mismatches are relatively small, making them suitable for constructing vdWHs. A smaller lattice mismatch promotes a tighter interface between the two materials, which is beneficial for optimizing electronic transport and optical properties. Therefore, these materials can be widely applied in the design and fabrication of vdWHs to achieve enhanced performance and application potential.

The stability of the structure of MPS_3_ MLs was explored through various methods initially. Phonon dispersion calculations were conducted to verify dynamic stability. As depicted in Figure 1b–d, no imaginary frequency modes were observed within the Brillouin zone for all 2D MPS_3_ MLs, confirming their dynamic stability. The absence of imaginary frequencies indicates that these materials do not exhibit any unstable vibrational modes that could lead to structural deformations or instabilities. This finding is essential as it assures the reliability and long-term viability of MPS_3_ MLs in practical applications. The computed bond length between M and M atoms (d_M-M_), the bond length between M and P atoms (d_M-P_), and the distance between P and X atoms (d_P-S_) for relaxed MPS_3_ MLs are shown in Table 1. In the MPS_3_ (M = Mn, Fe, and Ni) MLs, it is evident that the values of d_M-M_ decrease sequentially as 3.54 Å, 3.42 Å, and 3.39 Å. Additionally, the differences of d_P-S_ and d_P-P_ are about 2.00 Å and 2.04 Å (the differences between these three structures can be considered negligible). In the MPS_3_ MLs, a systematic reduction in the interatomic distance between M atoms was observed and this trend may be influenced by the varying electronegativities of these transition metal elements, affecting the strengths of their bonding interactions.

In pure GaN ML, the Ga−N bond length is 1.842 Å, while in vdWHs, it shortens to 1.822 Å, 1.813 Å, and 1.805 Å (shown in Table S1). The bond length reductions indicate strengthened Ga−N bonds in vdWHs, implying increased bond energy and enhanced bond stability. By examining Table 1, it is evident that under the influence of GaN ML, vdWHs experience lattice expansion. The lattice constants (a values) of these three vdWHs are found to be 6.42 Å, 6.39 Å, and 6.22 Å, respectively. This expansion leads to an increased distance between M atoms within the vdWHs. The observed lattice expansion can be attributed to the interlayer interactions between the GaN and MPS_3_ MLs. These interactions alter the equilibrium positions of the atoms, resulting in larger lattice constants and expanded unit cell sizes. The increased distance between M atoms implies a weakening of their interatomic interactions. Changes in interatomic spacing have significant implications for the electronic and structural properties of vdWHs, impacting their optical, electrical, and magnetic characteristics. It is worth noting that these changes extend beyond mere lattice expansion. Further investigation is warranted to fully comprehend the overall impact on the behavior and performance of vdWHs.

Binding energy (E_b_) represents the difference in the total energy of the vdWHs and their parent MLs as follows: E_b_ = E_total_ − E_MPS3_ − E_GaN_. Where E_total_, E_MPS3_, and E_GaN_ correspond to the energies of MPS_3_/GaN vdWHs, MPS_3_ and GaN MLs, respectively [40]. A positive E_b_ value indicates an endothermic reaction, where the energy of the system increases from reactants to products. Conversely, a negative E_b_ signifies an exothermic reaction, where energy is released as the system transitions from reactants to products. Our calculations reveal that the E_b_ of the vdWHs are −0.566 eV, −0.571 eV, and −0.591 eV, respectively (as shown in Table 1). The fact that all three vdWHs exhibit negative E_b_ values, which are also numerically similar, suggests that they possess similar binding strengths. The negative E_b_ indicates that energy is released during the formation of the vdWHs, implying that these BLs are thermodynamically stable. This stability is a crucial characteristic for the practical applications of vdWHs in fields such as optoelectronic devices, sensors, and catalysts. Moreover, the similar E_b_ values may imply structural and electronic property similarities among these vdWHs, which could be instructive for their performance in analogous applications.

### 3.2. Charge Analysis

**Figure 2:**
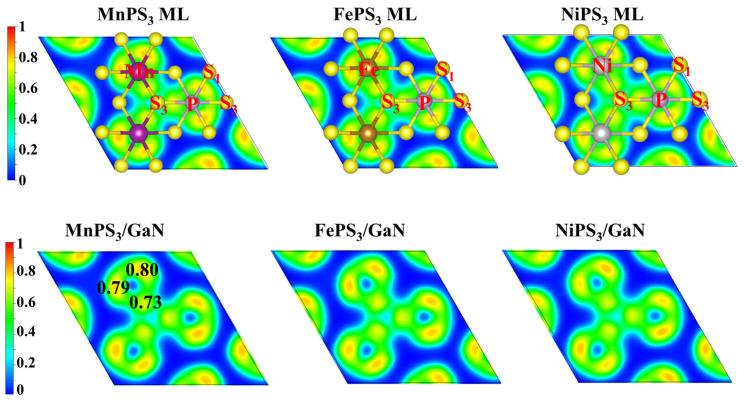
Top views of the ELF of the MPS_3_ MLs and vdWHs with the isosurface levels set at 0.45.

As shown in Figure 2, the Electron Localization Function (ELF) is commonly used to determine the bonding characteristics and the degree of electron localization, with its value ranging between 0 and 1. Here, ELF = 0 corresponds to the absence of electron distribution, while ELF = 0.5 represents a homogeneous electron gas. In contrast, ELF = 1 indicates complete electron localization at a specific site. Through computational analysis, it is found that both Mn and P atoms form bonds with S atoms, with ELF values ranging between 0.73 and 0.80. This suggests a strong covalent bonding interaction between these atoms, as ELF values closer to 1 signify higher electron localization and stronger covalent character. The ELF distribution around the bonding regions reveals a significant overlap of electron density between Mn–S and P–S, indicating the formation of stable chemical bonds. The relatively high ELF values (0.73~0.80) further support the conclusion that the electrons are highly localized in these bonding regions, which is characteristic of covalent interactions. Moreover, in the construction of the heterostructure bilayer, the ELF values remain consistently within this range (0.73–0.80). This indicates that the covalent bonding characteristics and electron localization behavior between Mn, P, and S atoms are preserved even in the heterostructure configuration. This analysis provides valuable insights into the electronic structure and bonding nature of the system, highlighting the importance of ELF in understanding the chemical properties and stability of the material.

To further analyze the electron distribution within the system, we employed the Bader charge analysis method. Bader analysis is a quantitative method based on the topological structure of the electron density. It can accurately partition the charge distribution between atoms and calculate the net charge carried by each atom. Through Bader analysis, we calculated the net charges of Mn, P, S, Ga, and N atoms and observed a distinct charge transfer phenomenon. As shown in Table 1, in the MPS_3_ MLs, M atoms and P atoms lose electrons, while S atoms gain electrons. This indicates that electrons are transferred from M atoms and P atoms to S atoms. This further confirms the polar characteristics of the M–S and P–S bonds, and this charge transfer behavior is consistent with the covalent bond characteristics observed in the ELF analysis. As the number of outermost electrons (M-d) increases, the ability of M atoms to lose electrons weakens. It has been calculated that the numbers of electrons lost by M atoms are 1.226 |e|, 1.085 |e|, and 0.825 |e|, respectively. This observation shows that as the number of outermost electrons increases and electron-donating ability decreases, which means that the tendency of M atoms to lose electrons is reduced. The numbers of electrons lost by P atoms are 0.952 |e|, 0.927 |e|, and 0.895 |e|, respectively, while the numbers of electrons gained by S atoms are 0.731 |e|, 0.676 |e|, and 0.580 |e|, respectively. These values indicate the extent of electron transfer between phosphorus atoms and sulfur atoms in the presence of different transition metals. The changes in the number of electrons lost by P atoms reflect the regulatory effect of different transition metals on their electron-donating ability. Similarly, the differences in the number of electrons gained by S atoms indicate their interactions with transition metals and the resulting charge redistribution. In contrast, in the MPS_3_/GaN vdWHs, the number of electrons lost by M atoms increases, the number of electrons lost by P atoms decreases, and the number of electrons gained by S atoms remains basically unchanged. As shown in Table S1, in pure GaN ML, Ga atoms lose 1.329 |e| while N atoms gain 1.329 |e|. In vdWHs, Ga atoms lose more charge (1.492 |e|, 1.509 |e|, and 1.522 |e|), and N atoms gain more charge (1.485 |e|, 1.502 |e|, and 1.513 |e|). This indicates significant electronic interactions between GaN and MPS_3_ layers in vdWHs, driving further charge transfer from Ga to N. The increased charge loss of Ga and gain of N in vdWHs versus pristine GaN show strengthened interlayer interactions, which enhance charge polarization within GaN, likely related to the electronic properties of the MPS_3_ layer.

This change can be attributed to the interaction between the MPS_3_ MLs and GaN, as well as the formation of the heterostructure. The interlayer interaction between MPS_3_ and GaN leads to the redistribution of electrons, with electrons flowing from MPS_3_ MLs to GaN MLs. The results of the Bader analysis provide valuable insights into the charge transfer behavior within the MPS_3_ MLs and the MPS_3_/GaN van der Waals heterobilayer system, highlighting the influence of interlayer interactions on electron redistribution and the resulting changes in electronic properties.

### 3.3. Electronic Properties

**Figure 3:**
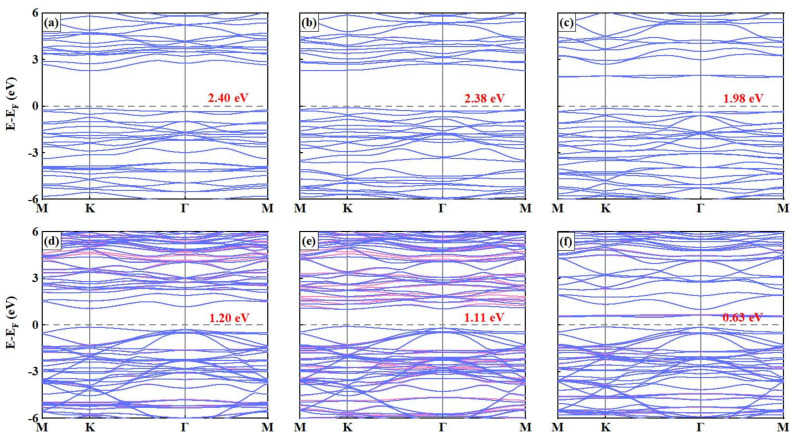
Band structure of MPS_3_ MLs and vdWHs: (**a**) MnPS_3_ MLs, (**b**) FePS_3_ MLs, (**c**) NiPS_3_ MLs, (**d**) MnPS_3_/GaN vdWs, (**e**) FePS_3_/GaN vdWs, and (**f**) NiPS_3_/GaN vdWs. The fermi level (EF) is marked in dashed lines. The bandgaps are listed in the corresponding pictures. The pink and blue lines represent the spin-up and spin-down channels, respectively.

**Figure 4:**
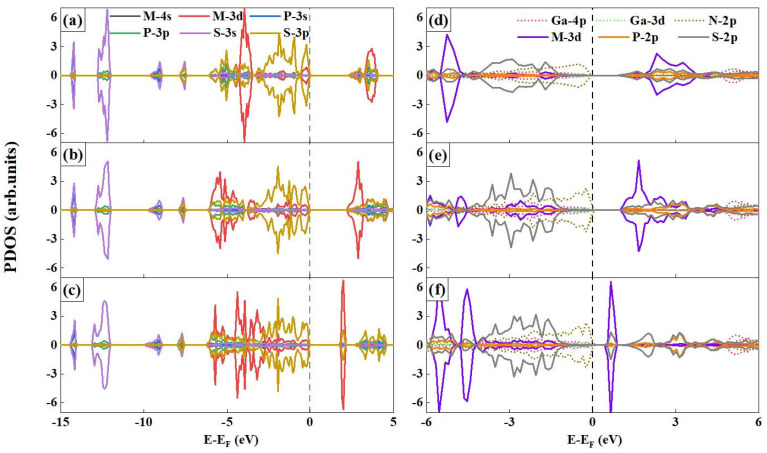
The projected density of states (PDOS) of MPS_3_ MLs and vdWHs: (**a**) MnPS_3_ ML, (**b**) FePS_3_ ML, (**c**) NiPS_3_ ML, (**d**) MnPS_3_/GaN vdWs, (**e**) FePS_3_/GaN vdWs, and (**f**) NiPS_3_/GaN vdWs. The Fermi level (EF) is marked in dashed lines.

Figure 3a–c presents the band structures of the MPS_3_ MLs, where it has been found that their ground states are AFM semiconductors with nearly degenerate energy bands in the spin-up and spin-down channels. Specifically, for the MnPS_3_ ML, having both the conduction band minimum (CBM) and the valence band maximum (VBM) located at the K point in the Brillouin zone indicates a direct band gap, meaning that electron transitions between the valence band and the conduction band can occur with minimal momentum change, whereas the FePS_3_ ML exhibits an indirect band gap with the CBM between the M and K points and the VBM solely at the K point. The NiPS_3_ ML, as an indirect band gap material with its CBM between the K and Γ points and the VBM remaining at the K point, has intermediate states of Ni-3d and S-3p within its energy gap that significantly reduce its band gap (as shown in Figure 4c), and in the indirect band gap of NiPS_3_ ML, the optical absorption or emission processes involve simultaneous changes in energy and momentum. We further confirmed these findings through the HSE06 method (shown in Figure S1, Supporting Materials), and understanding the band structure characteristics of these materials is crucial for tailoring their electrical, optical, and optoelectronic properties to meet specific application requirements. To gain a more in-depth understanding of their electronic properties, we calculated the projected density of states (PDOS) (results shown in Figure 4a–c), and the fact that the Fermi levels (E_F_) of the three MPS_3_ MLs structures overlap with the VBM indicates that these materials possess specific electronic properties such as a filled valence bands and low electrical conductivity. The S-3p and M-3d states were found to make significant contributions to the VBM, suggesting their crucial role in determining electronic behavior near the VBM, while the CBM is mainly occupied by the M-3d states, indicating that the electron transitions responsible for charge transport and conductivity involve the participation of these specific orbitals. Moreover, in the three MPS_3_ MLs, the tendency of the M-3d states to get closer to the E_F_ leads to a gradual decrease in the band gap, a phenomenon that can be attributed to the localized nature of the M-3d states playing an important role in narrowing band gaps as they increase the electronic density of states near the E_F_ and thus reduce the energy separation between the VBM and the CBM.

The electronic structure of vdWHs was investigated through comparative analysis of their constituent MPS_3_ MLs, revealing significant bandgap reductions of 50.00%, 53.16%, and 68.18% in MnPS_3_/GaN, FePS_3_/GaN, and NiPS_3_/GaN heterostructures, respectively, with corresponding BL bandgaps decreasing to 1.20 eV, 1.11 eV, and 0.63 eV compared to their MLs counterparts (2.40 eV, 2.37 eV, and 1.98 eV). This substantial bandgap narrowing arises from interfacial interactions and modified band alignment, where the EF shifts upward away from the VBM of MPS_3_ MLs, allowing GaN ML to dominate the VBM through its N atoms while the CBM remains governed by the antiferromagnetic MPS_3_ MLs, thereby establishing a type-II band alignment that spatially separates charge carriers across layers. Notably, MnPS_3_/GaN and FePS_3_/GaN vdWHs exhibit direct bandgaps with VBM and CBM localized at the K-point, whereas NiPS_3_/GaN vdWHs shows reversed alignment with Γ-point VBM and a CBM positioned between K-Γ points, with all systems demonstrating hybridization between GaN-derived N-2p and M-3d states at the interface. This unique electronic configuration facilitates the formation of interlayer excitons through spatial charge separation, suggesting enhanced potential for optoelectronic applications requiring efficient charge transfer mechanisms.

### 3.4. Optical Properties

The complex dielectric function is a crucial parameter for characterizing the optical properties of materials. It describes the linear response of materials to incident radiation (ε(ω) = ε_1_(ω) + iε_2_(ω)). Here, ε_1_(ω) and ε_2_(ω) represent the scattering of radiation within the material and the absorption of radiation by the material, respectively. The ε_2_(ω) spectrum can be calculated from the energy band dispersion, and ε_1_(ω) can be derived from ε_2_(ω) through the Kramers–Kronig transformation. Based on the complex dielectric function ε(ω), other optical functions can also be predicted, such as the absorption coefficient α(ω), reflectivity R(ω), and transmission T(ω) coefficient [41,42]. The optical absorption of MPS_3_/GaN vdW heterostructures is investigated by computing the complex dielectric function: ε(ω) = ε_1_(ω) + ε_2_(ω), where the imaginary part ε_2_(ω) is related to the absorption at a specific frequency ω, and the real part ε_1_(ω) is obtained from ε_2_(ω) using the Kramers–Kronig relation. The absorption spectrum can be calculated 1using the following formula:

$$\alpha \left(\omega \right)\;=\frac{2\omega }{\mathrm{ch}}{\left(\frac{\sqrt{\varepsilon_{1}^{2}+\varepsilon_{2}^{2}}-\varepsilon_{1}}{2}\right)}^{\frac{1}{2}}$$

where c is the speed of light in a vacuum. Strong anisotropy is observed in the absorption spectra α(ω) of MPS_3_ MLs and MPS_3_/GaN vdWHs along two polarization directions. The absorption coefficient is equal to the number of light leaps per unit volume.

**Figure 5:**
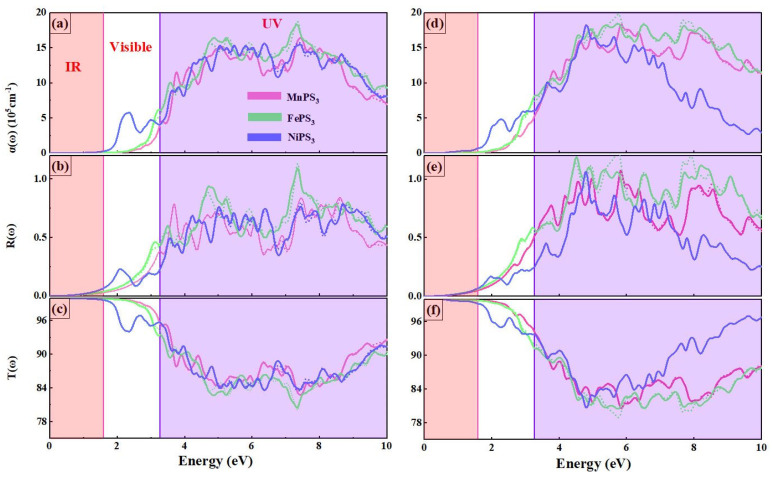
Calculated spectra of the (**a**) adsorption α(ω), (**b**) reflection R(ω), and (**c**) transmission T(ω) coefficients of MPS_3_ MLs and (**d**–**f**) for MPS_3_/GaN vdWHs.

Note that these spectra were calculated for incident radiation polarized parallel and perpendicular to the crystallographic directions. MPS_3_ MLs maintain optical isotropy in the specified energy range (0–3.29 eV), meaning that their absorption characteristics are uniform in all directions. This property is advantageous for applications requiring consistent optical performance, such as in optoelectronic devices. FePS_3_ and NiPS_3_ MLs display weak anisotropy at higher energies. This behavior can be attributed to their unique electronic structures and magnetic interactions, which lead to directional variations in optical absorption. From Figure 5a–c, it can be clearly observed that all three materials exhibit high sensitivity to the UV region and have a significant response intensity (on the order of 10^6^). This large response intensity indicates that these materials possess high-efficiency light absorption in the UV region, making them potentially valuable for UV detection or UV-based optoelectronic devices. In contrast, the absorption response of the NiPS_3_ ML is particularly prominent in the visible light region, with an absorption peak at around 2.3 eV. This suggests that NiPS_3_ ML has a smaller bandgap, enabling it to absorb visible light more effectively. This characteristic endows NiPS_3_ ML with a unique advantage in applications that require visible light absorption, such as in photovoltaics or photocatalysis. As shown in Figure 5d–f, the overall trend of the MPS_3_/GaN vdWHs curve exhibits a relatively weak change under the influence of the GaN ML, indicating that the GaN ML has a relatively minor impact on the electronic structure of the heterostructure. This may be due to the high stability and wide bandgap of the GaN ML itself, which limits its interaction with the host material. However, the red shift of the light absorption edge is a significant finding, indicating that the optical properties of the GaN ML have changed. This red shift is usually associated with a reduction in the bandgap, enabling the material to absorb photons with longer wavelengths. The introduction of the GaN ML opens up new possibilities for designing vdWHs with specific optical properties. By adjusting the bandgap and absorption range, researchers can optimize the performance of these materials in specific applications, such as improving the efficiency of photovoltaic devices or enhancing the performance of photocatalytic reactions.

### 3.5. Magnetic Properties

**Figure 6:**
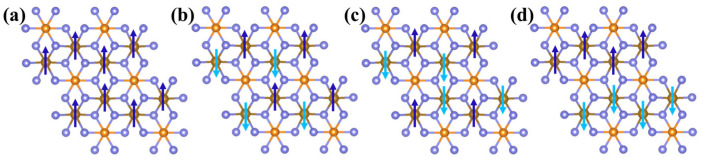
Geometric structures of four different magnetic configurations. (**a**) FM order, (**b**) Néel AFM order, (**c**) Stripy AFM order, and (**d**) Zigzag AFM order.

To further explore the magnetic mechanism of the system, 2 × 2 × 1 supercells were constructed (Figure 6) and four magnetic configurations were considered: (a) FM, (b) Néel AFM, (c) stripy AFM, and (d) zigzag AFM. It was found that in MnPS_3_ and NiPS_3_ MLs, the Néel-type antiferromagnetic order is energetically favored, while FePS_3_ exhibits a Zigzag-type antiferromagnetic order (as shown in Table 2). Specifically, the values for MPS3 MLs ground states are −279.73, −191.88, and −281.39 meV, respectively. These negative values unequivocally confirm the AFM nature of these structures. The magnetic moments contributed by each M (M = Mn, Fe, and Ni) atoms are 4.498, 3.672, and 1.517 $\mu_{B}$, respectively. Further testing revealed that the magnetic moments of these three structures are predominantly contributed by the M-3d states, accounting for 98.63%, 98.69%, and 99.87% of the total magnetic moment, respectively. This observation is closely related to the electronic configurations of transition metals (e.g., Mn: 3d^5^, Fe: 3d^6^, and Ni: 3d^8^), where the spin polarization of unpaired electrons in the 3d orbitals serves as the primary contributor to the magnetic moments, exhibiting strong localization characteristics (as shown in Figure 4d–f). Interestingly, in the vdWHs, the △E of the MPS_3_ MLs become −62.63, −54.30, and −55.21 eV, respectively. The magnetic moments of each M atom are 4.541 $\mu_{B}$ for Mn, 3.659 $\mu_{B}$ for Fe, and 1.489 $\mu_{B}$ for Ni. Upon forming vdWHs, the total energies decrease to −152.81, −90.39, and −178.82 eV, while the magnetic moments increase to 4.586, 3.677, and 1.564 $\mu_{B}$. The data show that compared to MPS_3_ MLs, the △E in vdWHs has increased by 45.37%, 52.89%, and 36.45%, respectively, while the MM has increased by 0.99%, 0.49%, and 5.04%. These changes indicate that the magnetic properties of the system have been significantly altered upon the formation of the van der Waals heterobilayer structure. To further validate their magnetic properties, we explored three distinct computational approaches: with and without considering van der Waals forces and the other with spin-orbit coupling (SOC) included (see Table S3). The results from both methods consistently align with the trends observed in the above analysis. The interlayer van der Waals interactions may induce hybridization between the M-3d orbitals and neighboring p/d states, thereby enhancing spin polarization. For instance, the 3d^8^ configuration of Ni demonstrates heightened sensitivity to interlayer charge transfer, resulting in the largest magnetic moment enhancement.

Moreover, the magnetocrystalline anisotropy energy (MAE) has been calculated to determine the preferred magnetization directions in MPS_3_ MLs [43,44,45]. This study investigated the magnetic orientation characteristics of MPS_3_ MLs and their vdWHs by calculating the MAE. Specifically, using the total energy difference between the in-plane [010] direction (corresponding to Cartesian axis b) and the out-of-plane [001] direction (corresponding to axis c) as the criterion (MAE = E_y_ − E_z_), the MAE values for the three types of MPS_3_ MLs were determined to be −0.33, 10.14, and 1.33 meV, respectively. Notably, two of the ML materials exhibited negative MAE values, indicating a lower energy advantage for the in-plane orientation. However, when configured into vdWHs MAE values significantly weakened, decreasing to −0.34, −3.59, and 0.15 meV, respectively. A sign reversal in the MAE of the second material group suggests that interlayer interactions may drive a transition in magnetic anisotropy from in-plane dominance to an out-of-plane preference. This comparative analysis highlights the regulatory role of interlayer coupling in two-dimensional materials on magnetic anisotropy, offering critical insights for the design of magnetic devices based on MPS_3_ systems.

## 4. Discussion

This study focuses on the two-dimensional antiferromagnetic materials MPS_3_ MLs (M = Mn, Fe, and Ni) and their vdWHs structures formed with GaN MLs, which have gradually attracted attention in the field of spintronics. Through first-principles calculations, we systematically studied the structural stability, charge transfer, band structure, density of states, optical properties, and magnetic properties of these materials, and explored their potential applications in optoelectronics and spintronics. By analyzing the phonon spectra, we confirmed the dynamic stability of the MPS_3_ MLs and conducted a detailed analysis of their bond lengths, charge distributions, and wide-bandgap semiconductor properties. In addition, based on the optical absorption spectra, reflectance, and transmittance, we explored the potential applications of the MPS_3_ MLs in the field of ultraviolet detection. The construction of the MPS_3_/GaN vdWHs led to a significant reduction in the bandgap (changing from 2.40, 2.38, and 1.98 to 1.20, 1.11, and 0.63), thus expanding the potential application range of its optical properties to the visible light region. Through calculations, we confirmed the intrinsic antiferromagnetic properties of the MPS_3_ MLs and determined the magnetic moments of the magnetic atoms M to be 4.560, 3.672, and 1.517 μB, respectively. The vdWHs structure further enhanced the magnetic moments of these elements. We conducted a further analysis of the magnetic properties of the MPS_3_ MLs and vdWHs using SOC and confirmed their magnetic anisotropy. These results provide a solid theoretical basis for the design of novel two-dimensional spintronic and optoelectronic devices based on MPS_3_.

## Figures and Tables

**Table 1:** The lattice parameter; energy difference (△E); binding energy (Eb); bond length between M and M atoms (dM-M); P and S atoms (dP-S); P and P atoms (dP-S) for relaxed MPS_3_ MLs and MPS_3_/GaN vdWHs; and the calculated charge transfer (CT) of M, P, and S atoms in MPS_3_ MLs and MPS_3_/GaN vdWHs.

	Lattice Parameters (Å)		Bond Length (Å)			CT (|e|)			△E (meV)	E _b_(eV)
	a	b	d_M-M_	d_P-S_	d_P-P_	M	P	S		
MPS_3_ MLs										
MnPS_3_	6.13	6.13	3.54	2.04	2.21	−1.191	−0.979	0.723	−59.57	−
FePS_3_	6.00	6.03	3.42	2.05	2.20	−1.065	−0.970	0.678	−52.51	−
NiPS_3_	5.89	5.89	3.39	2.04	2.18	−0.775	0.975	0.583	−55.21	−
vdWH BLs										
MnPS_3_	6.31	6.31	3.65	2.05	2.19	−1.226	−0.952	0.731	−44.81	−0.566
FePS_3_	6.27	6.27	3.59	2.06	2.18	−1.085	−0.927	0.676	−17.44	−0.571
NiPS_3_	6.24	6.23	3.60	2.07	2.16	−0.825	−0.895	0.580	−25.91	−0.591

**Table 2:** The energy difference △E-Néel (△E-N), △E-Stripy (△E-S), and △E-Zigzag (△E-Z) and the magnetic moment of per M atoms and M-d states in MPS_3_ MLs and MPS3/GaN vdWHs.

	MPS_3_ MLs			vdWHs		
M	Mn	Fe	Ni	Mn	Fe	Ni
△E-N (meV)	**−279.73**	−165.96	−238.98	**−152.81**	−68.56	−162.27
△E-S (meV)	−158.15	−40.30	59.49	−26.84	−34.16	41.95
△E-Z (meV)	−166.25	**−191.88**	**−281.39**	−102.59	**−90.39**	**−178.82**
M_M_ ($\mu_{B}$)	4.541	3.659	1.489	4.586	3.677	1.564
M_M-d_ ($\mu_{B}$)	4.479	3.611	1.487	4.524	3.627	1.558
MAE (meV)	−0.33	10.14	−1.33	−0.34	−3.59	0.15

## Data Availability

No data were used for the research described in the article.

## Supplementary Materials

The following supporting information can be downloaded at: https://www.mdpi.com/article/10.3390/nano15110832/s1.
