# Supplementary material for: Tunable Electronic Bandgaps and Optical and Magnetic Properties in Antiferromagnetic MPS3/GaN (M = Mn, Fe, and Ni) Heterobilayers
Source: Nanomaterials (Basel). 2025 May 30;15(11):832. doi: 10.3390/nano15110832 (PMC12156959; doi:10.3390/nano15110832)
Supplement: Supplementary file 1 [file nanomaterials-15-00832-s001.zip › nanomaterials-3629320-supplementary.pdf]

Tunable electronic bandgaps, optical and magnetic properties in antiferromagnetic MPS_3/GaN (M = Mn, Fe, Ni) heterobilayers

Shijian Tian[1,3,] Li Han[2*], Libo Zhang[3], Kuaixuan Zhang[3], Mengjie Jiang[1], Jie Wang[1], Shiqi Lan[1], Xuyang Lv[1], Yichong Zhang[1], Aijiang Lu[1], Yan Huang[4], Huaizhong Xing[1,*] and Xiaoshuang Chen[4]

1. The college of Physics, Donghua University, Shanghai 201620, China.
2. College of Optical and Electronic Technology, China Jiliang University, Hangzhou 310018, China.
3. College of Physics and Optoelectronic Engineering, Hangzhou Institute for Advanced Study, University of Chinese Academy of Sciences, No. 1, Sub-Lane Xiangshan, Xihu District, Hangzhou 310024, China.
4. State Key Laboratory of Infrared Physics, Shanghai Institute of Technical Physics, Chinese Academy of Sciences, Shanghai 200083, China.

*Correspondence: hanli0715@ucas.ac.cn (L.H.); xinghz@dhu.edu.cn (H.X.)

The differences in the calculated bandgap values of MPS_3 MLs using different methods primarily stem from disparities in theoretical frameworks, descriptions of electron interactions, and system adaptability. As a common functional within the GGA, the PBE algorithm corrects the LDA by incorporating electron density gradients. However, it still has limitations in describing the exchange-correlation energy of electrons in strongly correlated systems, particularly when handling localized d/p orbital electrons in MPS_3 MLs. By approximately neglecting part of the long-range exchange interactions, PBE underestimates the bandgap, yielding values of 2.40, 2.37, and 1.98 eV. In contrast, the HSE06 method, as a hybrid density functional, combines 25% exact Hartree-Fock exchange energy with correlation energy from the generalized gradient approximation. This allows it to more accurately characterize strong electron-electron interactions and the bandgap opening mechanism in energy band structures, especially suited for scenarios where electron localization is enhanced by quantum confinement effects in layered semiconductors. Additionally, HSE06 provides a more detailed treatment of charge transfer between atoms, orbital hybridization, and surface relaxation effects in MPS_3 MLs, effectively correcting the underestimation of bandgap caused by PBE's delocalization approximation and resulting in larger values: 3.68 eV, 4.33 eV, and 2.88 eV. Given that the HSE06 hybrid functional method requires incorporating partial exact Hartree-Fock exchange energy, leading to a computational complexity that increases exponentially with system size and k-point sampling density—thereby imposing significantly higher computational costs compared to the PBE functional within the GGA framework—the main text leverages the computational efficiency of the PBE method to analyze trends in bandgap variations.

Table S1. Charge transfer between Ga and N atoms in GaN monolayers and vdWHs, as well as the distance between Ga and N atoms ($d_{Ga–N}$).

	CT (|e|)		d_{Ga-N} (Å)
	Ga	N	
GaN ML	-1.329	1.326	1.842
vdWH-Mn	-1.492	1.485	1.822
vdWH-Fe	-1.509	1.502	1.813
vdWH-Ni	-1.522	1.513	1.805

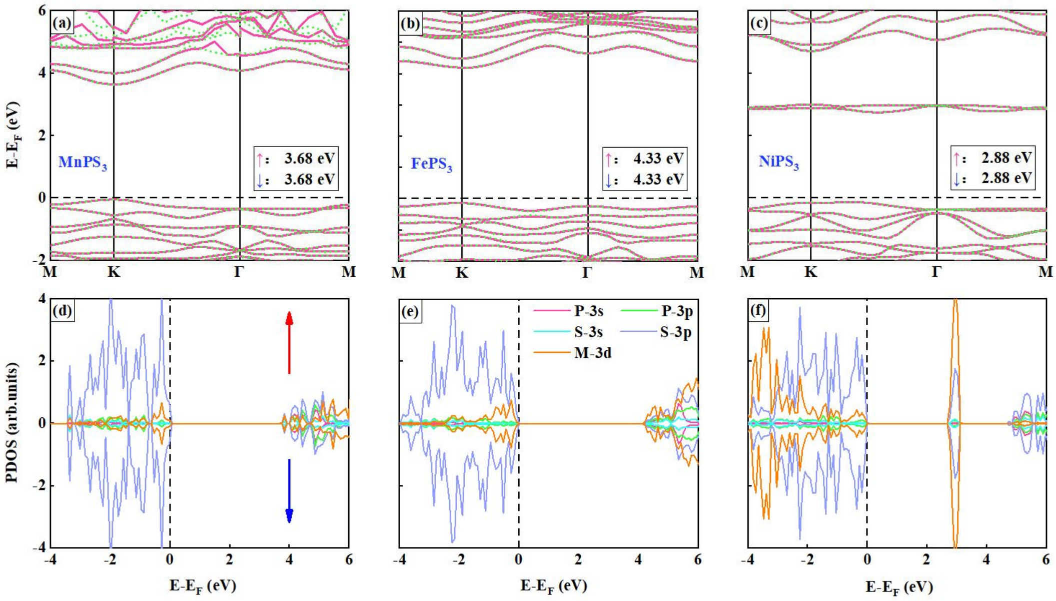

Fig. S1 Band structure of MPS_3 MLs calculated by HSE06 methods: (a) $MnPS_3$, (b) $FePS_3$ and (c) $NiPS_3$ MLs. The E_F is marked in dashed lines. The band-gaps are list in corresponding pictures.

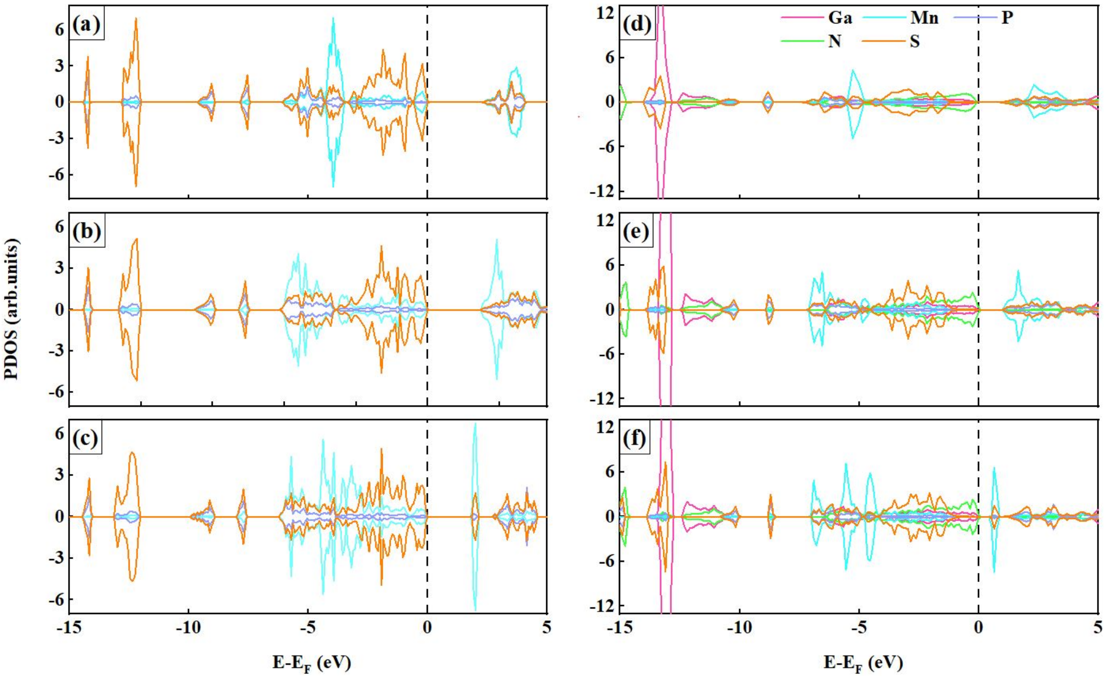

Fig. S2 LDOS of vdWHs bilayers in (a) $MnPS_3$/GaN bilayers, (b) $FePS_3$/GaN bilayers and (c) $NiPS_3$/GaN bilayers. The fermi level (E_F) is marked in dashed lines.

By setting the crystal relaxation control parameter ISIF=2 (fixing the unit cell volume while allowing atomic positions and cell shape relaxation) and ISIF=3 (fully

relaxing unit cell volume, shape, and atomic positions) in VASP, energy calculations were performed on the vdWHs formed by MPS_3 and GaN monolayers. The results show that the energy difference ΔE between the ferromagnetic (FM) and antiferromagnetic (AFM) configurations ($\Delta E = E_{FM} - E_{AFM}$) is negative under both conditions, indicating that the AFM state, with lower energy, constitutes the ground state of the system, consistent with the stability criterion for magnetic ordered structures. The negative E_b confirms that the formation of vdWHs from MPS_3 and GaN MLs is a thermodynamically spontaneous exothermic process accompanied by energy release. The consistent negative energy results under different relaxation conditions demonstrate the computational parameter robustness of the heterostructure's magnetic ground-state property and exothermic interfacial binding, providing theoretical support for the experimental fabrication of such vdWHs.

Table S2. The energy difference (△E), bindind energy (E_b), for relaxed MPS_3 MLs and MPS_3/GaN vdWHs BLs, The data calculated by three methods.

	MPS_3 MLs			vdWH BLs		
M	Mn	Fe	Ni	Mn	Fe	Ni
ISIF = 2, U = 4.0, 4.6, 5.1						
△E (meV)	-66.77	-33.19	-57.07	-52.76	-19.04	-30.21
E_b (eV)	-	-	-	-0.58	-0.60	-0.61
ISIF = 3, U = 4.0, 4.6, 5.1						
△E (meV)	-59.79	-33.92	-55.17	-45.01	-16.98	-34.19
E_b (eV)	-	-	-	-0.566	-0.570	-0.717
ISIF = 4, U = 4.0, 4.6, 5.1						
△E (meV)	-59.57	-52.51	-55.21	-44.81	-17.44	-25.91
E_b (eV)	-	-	-	-0.565	-0.571	-0.591

Table S3. The energy difference ΔE-Néel (ΔE-N), ΔE-Stripy (ΔE-S), ΔE-Zigzag (ΔE-Z), the magnetic moment of per M atoms and M-d states in MPS_3 MLs and MPS_3/GaN vdWH BLs. The data calculated by three methods.

	MPS_3 MLs			MPS_3/GaN vdWH BLs		
M	Mn	Fe	Ni	Mn	Fe	Ni
novdW, U_{eff}= 4.0, 4.6, 5.1						
△E-N	**-237.71**	-131.63	-220.43	**-172.93**	-64.93	-157.47
△E-S	-132.80	-111.98	53.11	-90.75	41.09	47.14
△E-Z	-141.93	**-239.11**	**-257.19**	-107.11	**-90.53**	**-169.71**
M_M	4.559	3.674	1.511	4.581	3.698	1.564
M_{M-d}	4.497	3.621	1.508	4.519	3.648	1.558
vdW, U_{eff}= 4.0, 4.6, 5.1						
△E-N	**-279.73**	-165.96	-238.98	**-152.81**	-68.56	-162.27
△E-S	-158.15	-40.30	59.49	-26.84	-34.16	41.95
△E-Z	-166.25	**-191.88**	**-281.39**	-102.59	**-90.39**	**-178.82**
M_M	4.541	3.659	1.489	4.586	3.677	1.564
M_{M-d}	4.479	3.611	1.487	4.524	3.627	1.558
vdW+SOC, U_{eff}= 4.0, 4.6, 5.1						
△E-N	-278.38	-172.49	-211.89	**-152.06**	-92.99	-162.36
△E-S	-157.70	-124.01	58.64	-26.34	-43.36	41.13
△E-Z	-157.78	**-213.47**	**-278.38**	-102.11	**-98.65**	**-177.35**
M_M	4.541	3.657	1.489	4.585	3.675	1.562
M_{M-d}	4.478	3.614	4.486	4.523	3.625	1.554
